# AGING-RELATED CHANGES IN MENTAL, PHYSICAL, AND COGNITIVE HEALTH: THE IMPACT OF THE RETIREMENT TRANSITION

**DOI:** 10.1093/geroni/igz038.2938

**Published:** 2019-11-08

**Authors:** Robert S Stawski, Kelly D Chandler

**Affiliations:** Oregon State University, Corvallis, Oregon, United States

## Abstract

Retirement is an important transition in later life, associated with changes in social roles. It is unclear, however, whether the retirement transition modifies aging-related changes in mental, physical, and cognitive health. Using data from the Health and Retirement Study, we examined changes in depressive symptoms, self-rated health, and memory prior to, at, and after the retirement transition among 6,830 participants (Ages=50-97, 58% female) assessed biennially up to 10 times from 1992-2010. Preliminary results indicate a sudden and significant increase in depressive symptoms and decreases in self-rated health and memory at the transition to retirement (ps<.05). These effects increased among individuals retiring at older ages (ps<.01). Further, aging-related increases in depressive symptomatology became faster after retirement (p<.01). Aging-related decreases in self-rated health and memory were unchanged by the transition. Discussion will focus on the contribution of transitions to understanding trajectories of mental, physical, and cognitive health in later life.

